# Increasing coverage of pediatric diarrhea treatment in high-burden countries

**DOI:** 10.7189/jogh.09.010503

**Published:** 2019-06

**Authors:** Kate Schroder, Audrey Battu, Leslie Wentworth, Jason Houdek, Chizoba Fashanu, Owens Wiwa, Rosemary Kihoto, Gerald Macharia, Naresh Trikha, Parth Bahuguna, Harkesh Dabas, Damien Kirchoffer, Lorna Muhirwe, Patricia Mucheri, Andrew Musoke, Felix Lam

**Affiliations:** 1 Clinton Health Access Initiative, Boston, Massachusetts, USA; 2 Clinton Health Access Initiative, Abuja, Nigeria; 3 Clinton Health Access Initiative, Nairobi, Kenya; 4 Clinton Health Access Initiative, New Delhi, India; 5 Clinton Health Access Initiative, Kampala, Uganda

## Abstract

**Background:**

Diarrhea is the second leading cause of infectious deaths in children under-five globally. Oral rehydration salts (ORS) and zinc could avert an estimated 93% of deaths, but progress to increase coverage of these interventions has been largely stagnant over the past several decades. The Clinton Health Access Initiative (CHAI), along with donors and country governments in India, Kenya, Nigeria, and Uganda, implemented programs to scale-up ORS and zinc coverage from 2012 to 2016. The programs sought to demonstrate that increases in pediatric diarrhea treatment rates are possible at scale in high-burden settings through a holistic approach addressing both supply and demand barriers. We describe the overall program model and the activities undertaken in each country. The overall goal of the paper is to share the program results and lessons learned to inform other countries aiming to scale-up ORS and zinc.

**Methods:**

We used a triangulation approach, using population-based household surveys, public facility audits, and private outlet surveys, to evaluate the program model. We used pre- and post-program population-based household survey data to estimate the changes in coverage of ORS and zinc for treatment of diarrhea cases in children under-five in program areas. We also conducted secondary analysis of Demographic and Health Surveys (DHS) and Multiple Indicator Cluster Surveys (MICS) surveys in surrounding regions and compared annual coverage changes in the CHAI-supported program geographies to the surrounding regions.

**Results:**

Across CHAI-supported focal geographies, the average ORS coverage across the program areas increased from 35% to 48% and combined ORS and zinc coverage increased from 1% to 24%. ORS coverage increases were statistically significant in the program states in India, from 22% (95% confidence interval CI = 21–23%) to 48% (95% CI = 47–50%) and program states in Nigeria, from 38% (95% CI = 32–40%) to 55% (95% CI = 51–58%). For combined ORS and zinc, coverage increases were statistically significant in all program geographies. Compared to surrounding regions, the estimated annual changes in combined ORS and zinc coverage were greater in program geographies. Using the Lives Saved Tool and based on the coverage changes during the program period, we estimated 76 090 diarrheal deaths were averted in the program geographies.

**Conclusions:**

Increasing ORS and zinc coverage at scale in high-burden countries and states is possible through a comprehensive approach that targets both demand and supply barriers, including pricing, optimal product qualities, provider dispensing practices, stocking rates, and consumer demand.

Diarrhea is the second leading cause of infectious deaths in children under-five globally, causing an estimated 526 000 deaths each year, or nearly 1500 childhood deaths each day [1]. While vaccines have the potential to prevent a considerable amount of diarrhea deaths, treatment is critical to addressing the rest. Effective, affordable treatment is available with oral rehydration salts (ORS) and zinc; treatment can prevent up to 93% of diarrhea deaths in children and costs less than US$0.50 per treatment course [2].

ORS was first introduced in the 1970s in Bangladesh by the International Centre for Diarrhoeal Disease Research, Bangladesh (icddr,b) and was championed globally as one of the most important medical advances of the 20^th^ century [3,4]. Usage rates of ORS steadily increased through the 1980s and early-1990s, driven by large-scale diarrhea control efforts by the World Health Organization (WHO), the United Nations International Children’s Emergency Fund (UNICEF), and many country government and international partners – contributing to significant reductions in diarrhea mortality globally. Despite these successes, global ORS coverage rates largely plateaued in the mid-1990s, and international focus and priorities shifted to other disease areas such as malaria and HIV. Global ORS treatment rates remained relatively flat through 2010 with roughly one in three children with diarrhea receiving ORS [5].

In 2004, the WHO and UNICEF recommended adjunct therapy with zinc alongside ORS as the primary treatment for childhood diarrhea [6]. In addition to averting deaths, systematic reviews of zinc have shown the therapy reduces the duration of illness and reoccurrence of diarrhea [7,8]. However, by 2012, zinc coverage was below 5% globally though in some countries such as Bangladesh, coverage was as high as 41% [9]. Many pilots had been completed to increase zinc and ORS usage, but often at sub-national levels [10-14]. For example, in Nepal, the Point-of-Use-Water Disinfection and Zinc (POUZN) project used radio and television and public and provider trainings to disseminate messages about appropriate diarrhea treatment and implemented targeted activities in 30 of 75 districts from 2005-2010. While caregivers who heard these messages were two times more likely to use zinc those who had not, national usage of zinc among pediatric diarrhea cases had only increased to 6% by 2011 [10,15,16]. Other pilots addressed only one of several potential barriers, such as provider knowledge and practices [11].

Bangladesh was the only country to achieve high treatment rates nationally: as of 2014 nearly 80% of children with diarrhea received ORS, and 34% received both ORS and zinc [17]. This was achieved through decades of investment by the government, icddr,b the international development organization BRAC, and other key stakeholders [18,19]. This effort included the large-scale Scaling Up Zinc for Young Children (SUZY) Project which was initiated in 2003 and included partnerships with the government, non-governmental organizations (NGOs), the private sector to improve provider dispensing, caregiver demand and availability of optimal, affordable products [20,21].

Building off the lessons from Bangladesh, POUZN, and other ORS and zinc scale-up efforts, such as the Strengthening Health Outcomes in Private Sector (SHOPS), the Clinton Health Access Initiative (CHAI) began partnerships with donors and governments in 2012 in four high-burden countries – India, Kenya, Nigeria, and Uganda – to take a comprehensive approach to ORS and zinc scale up. These efforts aimed to demonstrate that increased ORS and zinc coverage rates are achievable at scale in other high-burden settings beyond Bangladesh.

This paper describes the overall program model and the activities undertaken in each country. The overall goal of the paper is to share program results and lessons learned to inform other countries aiming to scale-up ORS and zinc.

## Program model

The theoretical underpinning of CHAI’s program model was that low demand for ORS and zinc was reinforced by low investment from manufacturers and suppliers, thus creating a “market trap” [22]. ORS and zinc had substantial commercial potential since there are more than 1.7 billion under-five diarrhea cases annually [23]. However, most manufacturers were not investing in widespread ORS and zinc distribution and promotion due to low product margins and demand. Many caregivers and health care providers were often unaware that ORS and zinc were the recommended treatments for diarrhea, and consequently did not demand or purchase these products [24].

The program’s theory of change (Figure S1 in **Online Supplementary Document**) sought to reduce child mortality by significantly increasing the percent of diarrhea cases treated with ORS and zinc. With donor support, the program worked to improve coverage rates in Kenya and Uganda, three states of India (Gujarat, Madhya Pradesh, and Uttar Pradesh), and eight states of Nigeria (Bauchi, Cross River, Kano, Katsina, Kaduna, Lagos, Niger, Rivers). These areas were selected based on high disease burden, low ORS and zinc coverage levels, and strong government buy-in. Kenya and Uganda have an estimated 5.9 million and 6.1 million number of children under five, respectively [25,26]. In Kenya and Uganda, diarrhea accounts for 7% of the 74 000 under-five deaths and 8% of the 85 000 under-five deaths, respectively [1]. Although the program worked in specific states of India and Nigeria rather than at national scale, the three program states in India have 33.3 million children under five and the eight program states in Nigeria have 8.1 million children under five [27,28]. In the three program states of India, diarrhea is estimated to be responsible for 8% of the 327 000 under five deaths [29]. In the eight program states of Nigeria, diarrhea is responsible for an estimated 37 502 under-five deaths [30].

To achieve increases in coverage, the program implemented a comprehensive model to break the market trap. As outlined below and summarized in **Table 1**, the program model addressed four major intervention areas:

**Table 1:** Overview of comprehensive program model and intervention areas

Intervention areas	Sample activities
Provider demand	Work with professional associations and governments to strengthen existing platforms that repeatedly reach public and private providers with education and mentorship
	Apply pharmaceutical industry techniques to change the practices of private providers through routine detailing, ie, promotional/sales visits
Supply availability	Engage local manufacturers and distributors to invest in production, promotion, and sales by providing supplier partners with market intelligence on projected demand and technical assistance on product registration, cost reduction, marketing, and product packaging
	Target wholesalers, sub-distributors, and retailers to expand the reach in hard-to-reach areas through innovative private sector strategies and streamlined distribution models
	Assist governments to access high-quality affordable products, as well as quantification, procurement, and distribution
Caregiver and consumer demand	Leverage networks that have the greatest reach, including mass media, religious schools, health talks at primary health centers, and community health workers
	Use private-sector best practices to develop consumer demand generation messages based on in-depth research of the most effective messages and channels to reach the target audience, specifically rural mothers with children under five.
Enabling environment	Work with governments and partners to align and to optimize diarrhea treatment scale-up efforts across stakeholders and integrate within existing child health services
	Secure over-the-counter status for zinc

- **Provider demand**: The program worked with professional associations and governments to strengthen existing platforms that repeatedly reach public and private providers with education and mentorship. In the private sector, the program applied pharmaceutical industry techniques to change the practices of providers through routine promotional/sales visits known as detailing.
- **Supply availability**: The program engaged local manufacturers and distributors to invest in production, promotion, and sales. The program provided interested high-quality suppliers with market intelligence on projected demand and also technical assistance on product registration, cost reduction, marketing, and product packaging. To expand the reach of ORS and zinc in hard-to-reach areas, innovative private sector strategies and streamlined distribution models were also pursued to target wholesalers, sub-distributors, and retailers. In the public sector, the program assisted governments to access high-quality, affordable products, as well as technical assistance with quantification, procurement, and distribution.
- **Enabling environment**: The program worked with governments and partners to align and to optimize diarrhea treatment scale-up efforts across stakeholders, to drive integration within existing child health services, and to secure over-the-counter status for zinc.
- **Consumer demand**: Caregivers were educated on diarrhea management, leveraging networks that had the greatest reach, including mass media, religious schools, health talks at primary health centers, and community health workers. In each country, consumer demand generation efforts were based on in-depth research of the most effective messages and channels to reach the target audience, specifically rural mothers with children under-five.

For each program geographical area, the specific activities undertaken were tailored to the local context in order to target the greatest barriers in that setting. For example, the relative emphasis on improving practices of public vs private providers was based on the underlying public and private sector care-seeking trends in that geography. **Table 2** summarizes the specific interventions used in each program area.

**Table 2:** Specific program activities by program country and intervention area

Program area	Provider demand	Supply availability	Enabling Environment	Consumer demand
India (Gujarat, Madhya Pradesh, Uttar Pradesh)	Public sector: supportive supervision to community health workers (ASHAs)	Public sector: improved product (flavored, dispersible, consumer-friendly packaging); better quantification to ensure consistent stock	Over-the-counter status for zinc	Mass media campaign in 2015 and 2016 on 25 national & regional channels
	Private sector: monthly detailing to ~ 130 000 providers; government-led education sessions	Private sector: streamlined rural supply chain with improved margins, rural stock points	National Intensified Diarrhea Control Fortnight	Interpersonal outreach in 2013-2014 with self-help groups and schools
	Job aids and ORS and zinc marketing materials for all providers		Updated diarrhea module in national IMCI materials	Consumer research on ORS and zinc packaging, messaging, and optimal channel mix
Kenya	Public sector: 5-day IMCI training of 4500+ health workers across 20 (of 47) counties with attendees required to train fellow facility staff to be certified	Public sector: MOH co-pack strategy, with bundled singles to prepare; forecasting support at national and county level	Over-the-counter status for Over-the-counter status for zinc; policy directive on co-pack switch	Government-led mass media campaign in 2014-2015 (program funded development of creative and government funded air time)
	Private sector: CMEs and routine provider detailing	Private sector: introduction of 3 locally produced co-packs	Government-led Essential Medicines scale up strategy that aligned efforts across partners	Daily “health talks” on key MNCH topics, including diarrhea, to caregivers at health centers
	Downloadable IMCI app; job aids and ORS and zinc marketing materials for all providers	Robust demand forecasts, product specifications, and MOH co-pack plans shared with suppliers	Updated diarrhea module in national IMCI materials	ORT corners at 1400 public facilities in 20 counties
Nigeria (Bauchi, Cross River, Kaduna, Kano, Katsina, Lagos, Niger, Rivers)	Public sector: leverage existing training platforms to reach over 75% of providers	Public sector: co-pack strategy by MOH; quantification support at national and state level	Over-the-counter status for zinc	Interpersonal outreach through Islamiyah schools, churches, key influencers
	Private sector: Repeated peer detailing of Proprietary Patent Medicine Vendors and chemists in partnership with their professional associations	Private sector: technical assistance to suppliers to facilitate new product introduction	Government-led Essential Medicines scale up strategy at national and state level that aligned efforts across all partners	Daily “health talks” on priority MNCH topics, including diarrhea, to caregivers waiting at health facilities
		Private sector: Supplier incentives to hit availability and price targets in rural areas; promotion at wholesale distributors	Updated diarrhea module in national and state IMCI materials	Radio campaign in 5 states (Bauchi, Kaduna, Kano, Katsina, Rivers)
Uganda	Public sector: CME on diarrhea management to providers in 35 (of 112) highest-burden districts	Public sector: MOH co-pack strategy; forecasting support at national and district level; ORS and zinc included in iCCM supply chain	Over-the-counter status for zinc	Radio campaign in 2014-2015 promoting zinc and ORS
	Private sector: 3-4 detailing visits to >75% of medicine outlets	Private sector: technical assistance to suppliers to facilitate new product introduction; promotion at wholesale distributors; recommended retail price	Government-led Essential Medicines scale up strategy that aligned efforts across all partners	Dissemination of ORS and zinc messages by 2800 CHWs through partnerships with BRAC, Living Goods, and World Vision
	SMS messages on ORS and zinc and job aids for all providers		Updated diarrhea module in national IMCI materials	

ORS – oral rehydration salts, CHW – community health workers, ASHA – Accredited Social Health Activist, IMCI – Integrated Management of Childhood Illnesses, MOH – Ministry of Health, CME – continuous medical education, MNCH – maternal, newborn, and child health, ORT – oral rehydration therapy, iCCM – integrated community case management, SMS – short message service

### Study objectives

The purpose of this study is to present program monitoring and evaluation results. The study focused on three research questions: (1) whether ORS and zinc coverage improved in program areas; (2) whether improvements in ORS and zinc coverage were equally observed among children living in rural areas and poor households; and (3) whether the annual rate of change in ORS and zinc coverage in program areas was greater than that in the surrounding region. Secondary study questions were whether availability of ORS and zinc changed during the program period, whether retail costs of ORS and zinc changed during the program period, and how many deaths were averted during the program period due to changes in ORS and zinc coverage.

## METHODS

### Data sources

We used a triangulation approach, using population-based household surveys, public facility audits, and private outlet surveys to evaluate the program model. Population-based household surveys were used to compare the percent of children under-five with diarrhea who received ORS and zinc before and after the program. The public health facility audits were used to check for the availability of ORS and zinc, and private outlet surveys were also used to track the market availability and price of ORS and zinc. We also estimated diarrhea deaths averted due to ORS and zinc coverage changes using Spectrum v5.753 Lives Saved Tool (LiST) (Avenir Health, Glastonbury, CT USA). The main paper focuses on results from the household surveys, and the methodology and results of the public facility audits, private outlet surveys, and LiST model are presented in the Online Supplementary Document. **Table 3** summarizes the data sources available for each program area.

**Table 3:** Data sources by program area and year

Program area	Household surveys	Public facility audits	Private outlet surveys
India (3 states*)	DLHS 2007-08	2013	2013
	CHAI 2014-15	2014-15	2014-15
	CHAI 2016	2016	2016
Kenya	DHS 2008-09	None	2013
	DHS 2014		2014
	KNBS/CHAI 2016		
Nigeria (8 states†)	CHAI 2013-14	2013-14	2013-14
	CHAI 2015	2015	2015
	CHAI 2016-17	2016-17	2016-17
Uganda	DHS 2011	2014	2014
	CHAI 2014	2015	2015
	DHS 2016	2016	2016

DLHS – district level household survey, CHAI – Clinton Health Access Initiative, DHS – Demographic and Health Survey, KNBS – Kenya National Bureau of Statistics

*India includes 3 states: Gujarat, Madhya Pradesh, and Uttar Pradesh.

†Nigeria includes 8 states: Bauchi, Cross River, Kaduna, Kano, Katsina, Lagos, Niger, Rivers.

For household surveys, we identified existing sources, such as Demographic and Health Surveys (DHS) and Multiple Indicator Cluster Surveys (MICS) that collected the necessary indicators prior to the program. In India, Kenya, and Uganda, the District Level Household Survey (DLHS) 2007-08, DHS 2008-09, and DHS 2011, respectively, were used for pre-program estimates of ORS and zinc coverage. In Nigeria, we hired an independent research agency to conduct a state-representative survey in the program states. For endline estimates, we hired independent agencies to conduct population-based household surveys in India, Kenya, and Nigeria. In Uganda, we conducted secondary analyses of the DHS 2016 survey. The sampling and survey methodologies for the program-funded household surveys were designed to be similar to the DHS. The methodology is described in detail elsewhere [31-33].

To compare the rate of change in ORS and zinc coverage in our program areas with the rates of change in the surrounding regions, we also searched for DHS and MICS surveys from countries in Sub-Saharan Africa, Asia, the Middle East, and Eastern Europe. Only countries which had at least two surveys conducted between 2008 and 2016 were included. All surveys included in the analysis are listed in **Table 4**.

**Table 4:** DHS and MICS surveys included in regional analysis

Country	Year	ORS and zinc coverage	Data source	Region
Ghana	2008	0.9	DHS 2008	SSA
Rwanda	2008	0.2	DHS 2008	SSA
Sierra Leone	2008	2.0	DHS 2008	SSA
Burundi	2010	0.1	DHS 2010	SSA
Chad	2010	0.2	MICS 2010	SSA
Democratic Republic of the Congo	2010	1.1	MICS 2010	SSA
Gambia	2010	0.0	DHS 2010	SSA
Malawi	2010	0.2	DHS 2010	SSA
Senegal	2010	0.1	DHS 2010	SSA
Sierra Leone	2010	0.9	MICS 2010	SSA
Eswatini	2010	0.0	MICS 2010	SSA
Togo	2010	0.3	MICS 2010	SSA
United Republic of Tanzania	2010	2.9	DHS 2010	SSA
Zimbabwe	2010	0.0	DHS 2010	SSA
Benin	2011	8.7	DHS 2011	SSA
Cameroon	2011	0.0	DHS 2011	SSA
Côte d'Ivoire	2011	0.1	DHS 2011	SSA
Ethiopia	2011	0.0	DHS 2011	SSA
Ghana	2011	0.0	MICS 2011	SSA
Mauritania	2011	0.2	MICS 2011	SSA
Guinea	2012	0.3	DHS 2012	SSA
Mali	2012	1.4	DHS 2012	SSA
Senegal	2012	0.4	DHS 2012	SSA
Democratic Republic of the Congo	2013	1.6	DHS 2013	SSA
Gambia	2013	0.0	DHS 2013	SSA
Malawi	2013	23.0	MICS 2013	SSA
Sierra Leone	2013	3.4	DHS 2013	SSA
Togo	2013	0.1	DHS 2013	SSA
Benin	2014	15.5	MICS 2014	SSA
Cameroon	2014	5.2	MICS 2014	SSA
Ghana	2014	5.5	DHS 2014	SSA
Senegal	2014	0.7	DHS 2014	SSA
Eswatini	2014	42.3	MICS 2014	SSA
Zimbabwe	2014	13.8	MICS 2014	SSA
Chad	2014-15	0.5	DHS 2014-2015	SSA
Rwanda	2014-15	7.0	DHS 2014-2015	SSA
Mali	2015	2.3	MICS 2015	SSA
Mauritania	2015	16.0	MICS 2015	SSA
Senegal	2015	7.2	DHS 2015	SSA
Zimbabwe	2015	14.9	DHS 2015	SSA
Malawi	2015-16	24.4	DHS 2015-2016	SSA
United Republic of Tanzania	2015-16	13.4	DHS 2015-16	SSA
Côte d'Ivoire	2016	5.6	MICS 2016	SSA
Ethiopia	2016	17.0	DHS 2016	SSA
Guinea	2016	16.3	MICS 2016	SSA
Senegal	2016	4.9	DHS 2016	SSA
Burundi	2016-17	6.0	DHS 2016-17	SSA
Philippines	2008	1.2	DHS 2008	Asia/M. East/E. Europe
Afghanistan	2010	4.3	MICS 2010	Asia/M. East/E. Europe
Cambodia	2010	0.9	DHS 2010	Asia/M. East/E. Europe
Mongolia	2010	0.2	MICS 2010	Asia/M. East/E. Europe
Viet Nam	2010-11	0.8	MICS 2010-11	Asia/M. East/E. Europe
Nepal	2011	4.8	DHS 2011	Asia/M. East/E. Europe
Kyrgyzstan	2012	0.0	DHS 2012	Asia/M. East/E. Europe
Mongolia	2013	7.1	MICS 2013	Asia/M. East/E. Europe
Philippines	2013	4.6	DHS 2013	Asia/M. East/E. Europe
Viet Nam	2013	12.6	MICS 2013	Asia/M. East/E. Europe
Cambodia	2014	3.2	DHS 2014	Asia/M. East/E. Europe
Kyrgyzstan	2014	8.6	MICS 2014	Asia/M. East/E. Europe
Nepal	2014	18.2	MICS 2014	Asia/M. East/E. Europe
Afghanistan	2015	7.1	DHS 2015	Asia/M. East/E. Europe
Nepal	2016	10.3	DHS 2016	Asia/M. East/E. Europe
Bangladesh	2011	33.0	DHS 2011	Bangladesh
Bangladesh	2012-13	10.6	MICS 2012-13	Bangladesh
Bangladesh	2014	35.9	DHS 2014	Bangladesh
Timor Leste	2009	4.1	DHS 2009	Timor Leste
Timor Leste	2016	40.0	DHS 2016	Timor Leste

DHS – Demographic and Health Survey, MICS – Multiple Indicator Cluster Survey, ORS – oral rehydration salts, SSA – Sub-Saharan Africa, M. East – Middle East, E. Europe – Eastern Europe

### Statistical analyses

To determine whether ORS and zinc coverage changed in our program areas, we estimated ORS and combined ORS and zinc coverage and corresponding 95% confidence intervals for each survey conducted in our program area while applying the appropriate sampling weights for the respective survey. We consider estimates with non-overlapping confidence intervals to be statistically significant.

To determine whether ORS and zinc coverage changes were equally observed among rural and poor households, we estimated ORS and zinc coverage by urban/rural and lowest/highest household wealth quintiles for each survey conducted in our program areas. Definitions of urban and rural areas were defined by the sampling frames used for those respective survey and wealth quintiles were constructed using principle component analysis of household assets [31-38].

We summarized the baseline and endline estimates using unweighted averages across program areas to provide a measure of overall program coverage change. Unweighted averages were also used to summarize baseline and endline coverage levels across program areas by urban/rural areas and wealth. The survey analyses were conducted in Stata 14 (StataCorp, College Station, TX, USA).

To determine whether coverage changes in program areas were greater than those in the surrounding region, we estimated the rate of change for ORS and zinc coverage in percentage points per year using ordinary least squares method for two periods: a pre-program period between 2008 and 2012 and a program period between 2012 and 2016. Four-year time periods were used as the program periods in the countries were approximately four years. We estimated annual coverage changes between 2008 and 2012 to determine whether pre-program trends were similar between geographies and between 2012 and 2016 to determine whether ORS and zinc coverage changes in program areas were greater during the program period. We first disaggregated the surveys conducted between 2008-12 and 2012-16. Surveys conducted in 2012 were included in both sets. We calculated coverage levels for the years 2008 and 2012 if survey data was not available for those years. These estimates were achieved by calculating the average change in coverage between the two available data points immediately before and after 2008, then applying this linear trend to estimate coverage in 2008 and then again in 2012. Using this method, data points for 2008 and 2012 were calculated for all countries and program areas to fill in these missing points.

We then plotted combined ORS and zinc coverage for the year in which the survey was conducted and estimated a best fit line using ordinary least squares. We conducted the analysis separately for the individual program areas, the Sub-Saharan Africa region, and the Asia/Middle East/Eastern Europe region. Countries in Asia, Middle East, and Eastern Europe were grouped together given scarcity of data in the individual regions.

## RESULTS

### ORS and zinc coverage in program areas

**Table 5** presents coverage estimates before and after the program. On average, ORS coverage increased by 13 percentage points – from 35% to 48% – across the four program areas. We found statistically significant ORS coverage increases in India and Nigeria. In India, ORS coverage in the program states increased from 22% (95% CI = 21-23%) to 48% (95% CI = 47-50%). In Nigeria, ORS coverage in program states increased from 38% (95% CI = 34-42%) to 55% (95% CI = 51-58%).

**Table 5:** Percent of children 0-59 months receiving ORS and zinc for treatment of diarrhea in the last 2 weeks

	ORS coverage		Combined ORS+Zinc coverage	
**Program area**	**Pre-program (95% CI)**	**Post-program (95% CI)**	**Pre-program (95% CI)**	**Post-program (95% CI)**
India (3 states*)	22.2 (21.3-23.1)	48.4 (46.8-50.0)	0.0	19.4 (18.1-20.7)
Kenya	38.8 (34.0-43.8)	42.2 (37.8-46.7)	0.2 (0.0-0.8)	15.2 (11.9-19.2)
Nigeria (8 states†)	37.9 (34.4-41.5)	54.7 (51.2-58.1)	3.7 (2.7-5.0)	30.0 (27.1-33.0)
Uganda	43.5 (39.8-47.4)	46.8 (44.6-49.1)	1.2 (0.7-2.0)	29.6 (27.5-31.8)
Average‡	35.0	48.0	1.1	23.8

ORS – oral rehydration salts, CI – confidence interval

*India includes 3 states: Gujarat, Madhya Pradesh, and Uttar Pradesh.

†Nigeria includes 8 states: Bauchi, Cross River, Kaduna, Kano, Katsina, Lagos, Niger, Rivers.

‡Unweighted average across program geographies.

For combined ORS and zinc coverage, we found statistically significant coverage changes in all four program areas, and on average, ORS and zinc coverage increased by 23 percentage points. In program states of India, combined ORS and zinc coverage increased from 0% to 19% (95% CI = 18-21%). In Kenya, ORS and zinc coverage increased from 0.2% (95% CI = 0-1%) to 15% (95% CI = 12-19%). In Nigeria program states, combined coverage increased from 4% (95% CI = 3-5%) to 30% (95% CI = 2733%). In Uganda, ORS and zinc coverage increased from 1% (95% CI = 1-2%) to 30% (95% CI = 28-32%).

### ORS and zinc coverage in rural areas and among the poor

**Figure 1:**
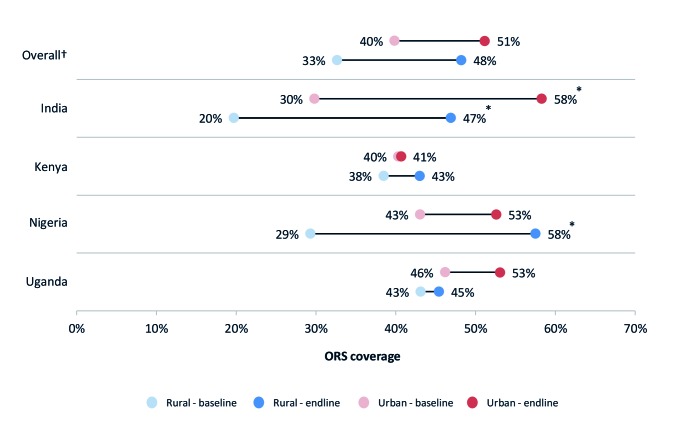
ORS coverage by rural/urban areas. *95% confidence intervals (CI) do not overlap. †Overall figures are unweighted averages of the four program geographies.

**Figure 2:**
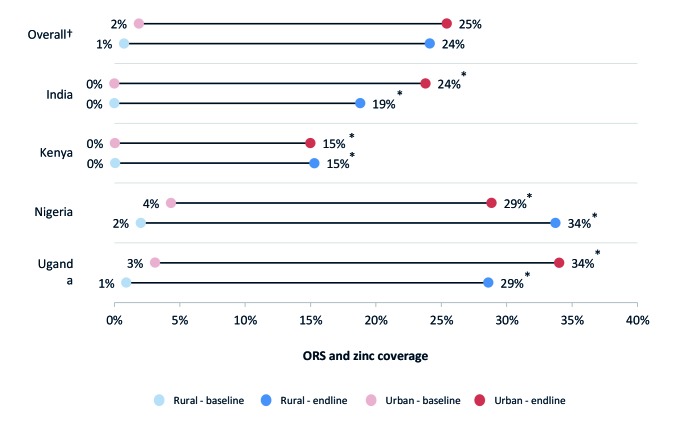
ORS and zinc coverage by rural/urban areas. *95% confidence intervals (CI) do not overlap. †Overall figures are unweighted averages of the four program geographies.

**Figure 3:**
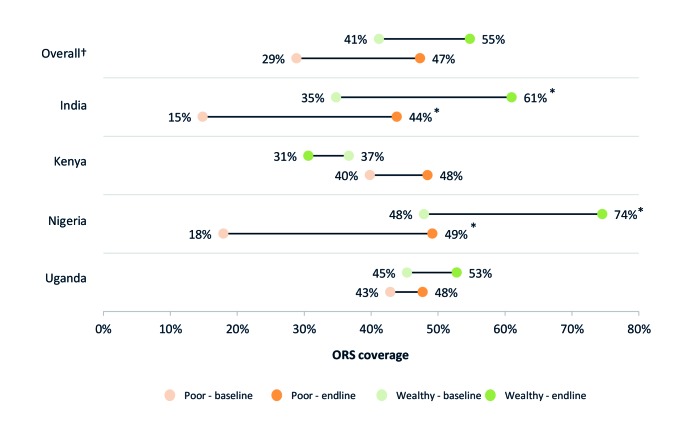
ORS coverage by wealth quintile (top 20% and bottom 20%). *95% confidence intervals (CI) do not overlap. †Overall figures are unweighted averages of the four program geographies.

**Figure 4:**
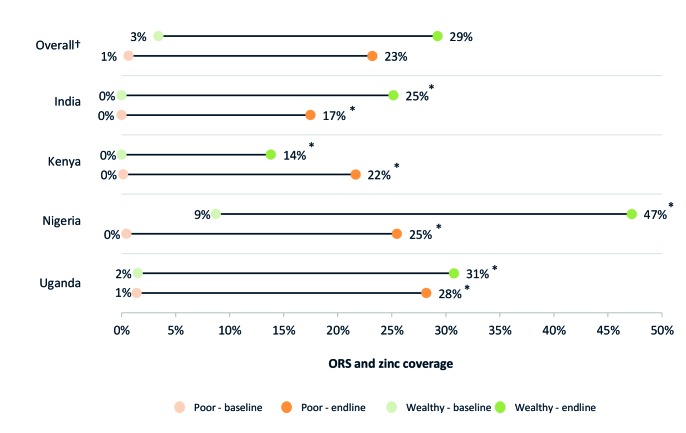
ORS and zinc coverage by wealth quintile (top 20% and bottom 20%). *95% confidence intervals (CI) do not overlap. †Overall figures are unweighted averages of the four program geographies.

**Figure 1** and **Figure 2** present ORS and combined ORS and zinc coverage changes, respectively, in urban and rural households. **Figure 3** and **Figure 4** present ORS and combined ORS and zinc coverage changes, respectively, in the poorest and wealthiest quintiles. We found coverage increased in both rural and urban areas and across wealth quintiles. On average across the four program areas, ORS coverage increased from 40% to 51% in urban areas – a relative increase of 28% – while in rural areas, ORS coverage increased on average from 33% to 48% – a relative increase of 48%. Likewise among the poorest quintile, ORS coverage increased across the four program areas on average from 29% to 47% – a relative increase of 64% – while coverage in the wealthiest quintile increased from 41% to 55% – a relative increase of 33%.

In India and Nigeria, we found a statistically significant increase in ORS coverage for children with diarrhea living in rural areas. In India, ORS coverage in rural areas increased from 20% (95% CI = 19-21%) to 47% (95% CI = 45-49%). In Nigeria, the percent of diarrhea cases living in rural areas receiving ORS increased from 29% (95% CI = 25-34%) to 58% (95% CI = 54-61%). We also found coverage in urban areas in India had increased significantly, though the relative change in rural areas were greater. In India, ORS coverage in rural areas increased by 138% compared to 96% in urban areas. The coverage increase in urban areas of Nigeria was not statistically significant: 43% (95% CI = 34-52%) at baseline and 53% (95% CI = 47-58%) at endline.

Among diarrhea cases living in the poorest quintile, we found ORS coverage had significantly increased in India and Nigeria. In India, ORS coverage in the poorest quintile increased by 29 percentage points, from 15% (95% CI = 14-16%) to 44% (95% CI = 42-46%). In Nigeria, the increase was by 31 percentage points, from 18% (95% CI = 21-26%) to 49% (95% CI = 42-57%).

### Comparison of program areas and other regions

**Table 6** presents estimates of annual ORS and zinc coverage change prior to the program (2008-2012) and during the program (2012-2016). Between 2008 and 2012, the Asia/Middle East/Eastern Europe region saw zero change in ORS and zinc coverage, while the program states in India saw coverage increase by 1.2 percentage points per year. Between 2012 and 2016, the Asia/Middle East/Eastern Europe region experienced average annual coverage increases of 1.7 percentage points per year, while we found average annual coverage increases of 2.5 percentage points per year in the program states in India.

**Table 6:** Comparison of annual coverage increases of combined ORS and zinc between program areas and regional averages by period 2008-2012 and 2012-2016

Program area/ Comparison region	ORS and zinc annual coverage increase between 2008-2012 (percentage points per year)	ORS and zinc annual coverage increase between 2012-2016 (percentage points per year)
**India (3 states) compared to Asia/Middle East/Eastern Europe:**		
Asia/Middle East/Eastern Europe*	0.0	1.7
India (3 states†)	1.3	2.5
**Nigeria (8 states), Uganda, Kenya compared to Sub-Saharan Africa:**		
Sub-Saharan Africa‡	0.1	2.2
Nigeria (8 program states§)	0.5	7.2
Uganda	0.2	7.1
Kenya	1.2	2.6
Bangladesh¶	3.7	4.6
Timor-Lest¶	4.9	5.1

ORS – oral rehydration salts

*Asia/Middle East/E Europe includes data from 7 countries (not including India as this was a program area or Bangadesh or Timor Leste as these were significant outliers for the region).

†India includes 3 states: Gujarat, Madhya Pradesh, and Uttar Pradesh.

‡Sub-Saharan Africa includes data from 21 countries (not including Nigeria, Uganda, or Kenya as these were program areas).

§Nigeria includes 8 program states: Bauchi, Cross River, Kaduna, Kano, Katsina, Lagos, Niger, and Rivers.

¶Bangladesh & Timor-Leste shown separately to illustrate the remarkable progress and that they are global outliers on ORS and zinc coverage.

We present Bangladesh separately given its unique experience in leading research and implementation of ORS and zinc scale-up. Timor-Leste is also presented separately as the coverage increase over the period of interest is a clear outlier for the region. We find average annual ORS and zinc coverage increases in Bangladesh and Timor-Leste was nearly equal in both periods. Between 2008 and 2012, ORS and zinc coverage increased at a rate of 3.7 and 4.9 percentage points per year respectively, and between 2012 and 2016, the coverage rate increase was 4.6 and 5.1 percentage points per year, respectively. The latest surveys show that combined ORS and zinc coverage in Bangladesh and Timor Leste are 36% and 40%, respectively.

In Sub-Saharan Africa, ORS and zinc coverage increased at an average annual rate of 0.1 percentage points per year between 2008 and 2012. Between 2012 and 2016 in Sub-Saharan Africa, the average annual ORS and zinc coverage increased by 2.2 percentage points per year. In Kenya, Uganda, and program states in Nigeria, we found average annual coverage increases of 1.2, 0.2, and 0.5, percentage points per year, respectively, between 2008 and 2012. Between 2012 and 2016, average annual coverage increased by 2.6 percentage points per year in Kenya, 7.1 percentage points per year in Uganda, and 7.2 percentage points per year in program states in Nigeria.

### Estimated diarrheal deaths averted due to ORS and zinc scale-up

Using the LiST model, we estimated 76 090 (sensitivity bounds 60 690-89 140) diarrheal deaths were averted during the program period due to increases in ORS and zinc coverage (Table S1 in **Online Supplementary Document**). We estimated the largest number of diarrheal deaths averted in the three program states of India: 48 030 (sensitivity bounds 38 590-56 090). In Nigeria’s eight program states, we estimated 18 160 (sensitivity bounds 14 810-20 920) diarrheal deaths were averted. In Kenya and Uganda, we estimated 3340 (sensitivity bounds 2670-3920) and 6560 (sensitivity bounds 4620 – 8210), respectively, diarrheal deaths averted.

## DISCUSSION

Our study findings suggest the program model demonstrated increased uptake of ORS and zinc at scale in focal geographies. National and statewide ORS and zinc coverage in program areas increased during the program period, and rates of coverage increases were greater than those in comparable regions. Results from the facility audits and private outlet surveys also show that ORS and zinc availability had also increased from an average of 57% to 79% in public facilities and 28% to 66% in private outlets, and prices on average declined by 42% from US$1.50 to US$0.87 (Figures S2-S6 in **Online Supplementary Document**). The concurrent changes in these indicators were consistent with the program’s theory of change which expected that improvements in product availability, pricing, provider dispensing, consumer demand and policy would likely contribute to overall improvements in population-level coverage rates (Figure S1 in **Online Supplementary Document**).

Bangladesh’s success was not an anomaly. Lessons of successful scale-up of diarrhea treatment rates in Bangladesh and these four additional high-burden geographies can provide a roadmap for broader scale up globally. While each country must adapt the interventions to their unique context, the evidence from this program suggests that the four core strategy components (provider demand, supply availability, enabling environment, consumer demand) can be consistent across countries. Bringing this approach to additional high-burden countries could reach millions more cases and save the lives of hundreds of thousands of more children. Alongside continued scale up of vaccines, improved diarrhea treatment rates could prevent the deaths of more than 500 000 children who die unnecessarily each year from diarrhea-related causes [39]. Summaries of the approach and lessons learned in each country context are described in **Boxes 1**** to ****4**.

Box 1 **Program summary and lessons learned in program states in India**

***Approach*:** In program states in India, ~ 70% of diarrhea cases seek care, with over 80% in the private sector. Given this, the program focused heavily on changing stocking and dispensing practices of rural medical providers (RMPs). Price was not a major barrier since the price was regulated in the private sector, and ORS and zinc were free in the public sector. To change RMP practices, the program partnered with rural entrepreneurs, who promoted and sold ORS and zinc in their villages, and connected them with quality regional suppliers. This model increased product margins by reducing supply chain handoffs and created greater flexibility in supply chain. The purchase of products was fully funded by the partners, while the program provided in-kind promotional materials and time-limited operations support. After three and a half years, the partners were fully self-sustaining and continued selling ORS and zinc as part of a broader basket of goods. In addition, CHAI partnered with government on diarrhea “orientation sessions” to educate RMPs on correct treatment. In the public sector, the program worked with state governments to optimize product specifications and packaging in response to consumer research. The program also launched an intensive mass media campaign in years 3 and 4 based on consumer research on most effective messages and channels.

***Mistakes/Challenges:*** The approach to consumer demand in years 1-2 focused on interpersonal outreach but shifted to mass media in years 3-4 as cost per contact was lower.

***Critical Success Factors:***

- Robust consumer research informed program design
- High media penetration even in rural and poor households
- Large-scale, self-sustaining rural sales force created platform for detailing, better margins, closer stock points

Box 4 **Program summary and lessons learned in Uganda**

***Approach***: In Uganda, ~ 70% of pediatric diarrhea cases seek care with ~ 45% in public sector and 55% in private sector (DHS 2011). At the start of the program, Uganda had a monopolistic, low-volume, high-price and often subsidized market with only two zinc and two ORS suppliers. To address this, the program provided TA to suppliers to bring new products to market, increasing competition to reduce prices. From these efforts, four low-osmolarity ORS, four dispersible zinc tablets, and two co-packaged products entered the market; average retail price declined ~ 40% to $0.71. The government had a co-pack strategy, and CHAI supported the government nationally and in all 112 districts to ensure volumes procured met public sector demand. In the supply chain, the program promoted zinc/ORS at wholesale distribution points and provided incentives to local distributors hitting availability and price targets (below recommended retail price which was introduced by government in late 2014.) To generate demand, CHAI provided promotional materials to suppliers, and supported professional associations and the MOH to incorporate updated guidelines into all existing CMEs, mentorship, and training platforms. These efforts reached >20K public and private providers. The program also worked through village health teams and community groups (BRAC, Living Goods) to promote zinc/ORS.

***Mistakes/Challenges:*** Initial introduction of the co-pack in the public sector occurred too soon – before affordable co-packs were on the market. This led to an initial co-pack tender that was more expensive than singles. With CHAI support, MOH renegotiated the co-pack tender price once affordable co-packs were available on the market to achieve prices below that of singles.

***Critical Success Factors:***

- Quantification/forecasting support in all 112 districts
- Co-pack strategy and efforts at all points in supply chain to ensure availability and competitive pricing

While combined ORS and zinc use increased in program areas, coverage remains relatively low: below 30% in each country. Efforts to further improve care-seeking for diarrhea and use of ORS and zinc should be continued. Diarrhea remains a major cause of mortality in these countries, and ORS and zinc are proven cost-effective interventions to reduce mortality in children under five. In Kenya and Uganda, approximately 30% of children with diarrhea still do not seek care and are therefore unlikely to receive ORS and zinc compared to those that seek care. Efforts to improve care-seeking in these countries could have an outsized effect in improving coverage. In India and Nigeria, coverage amongst cases seeking care in the private sector lags behind those in the public sector, and a higher proportion of children with diarrhea seek care in the private sector in these countries. Continuing to engage the private sector and encourage appropriate case management with ORS and zinc could help further gains in India and Nigeria.

### Supply availability and pricing

The program aimed to first ensure consistent availability of affordable, quality ORS and zinc products prior to investing in demand generation. At the start of the program, access to ORS and zinc was limited in Kenya, Nigeria, and Uganda, largely driven by a small number of expensive products on the market. There were no pediatric zinc products available on the market in Nigeria, only one in Kenya, and two in Uganda. The average cost of the full treatment course of ORS and zinc was US$1.67 in Nigeria, US$1.55 in Kenya, and US$1.27 in Uganda (Table S2 in **Online Supplementary Document**). By comparison, there were many ORS and zinc products already available on the market in India, and prices were regulated by the government.

To address availability and pricing challenges in the three African markets, CHAI and partners facilitated the entry of over 15 new ORS and zinc products in Kenya, Nigeria, and Uganda, including several local manufacturers. Suppliers were engaged through government-led supplier forums as well as in-kind technical assistance on product registration, costing, marketing, and packaging. The program did not subsidize or conduct free distribution of ORS and zinc in order to ensure sustainability of the interventions. Rather, the program found that providing suppliers with realistic demand projections and transparency into government scale-up plans helped to increase suppliers’ willingness to invest in market entry. In addition, CHAI worked in all four countries to improve availability in rural areas by connecting high-quality suppliers with rural distributors and wholesalers to enhance supply chain efficiency and stocking rates at rural outlets. Across the focal geographies, average availability of both zinc and ORS increased from 57% to 79% in public sector facilities (Figures S2 and S3 in **Online Supplementary Document**). In private-sector outlets, availability of both products increased from 28% to 66% (Figures S4 and S5 in **Online Supplementary Document**).

Three countries, Kenya, Nigeria, and Uganda, pursued a co-pack strategy in the public sector. This likely contributed to the rapid increase in combined coverage rates in these countries, though India also increased ORS and zinc coverage from 0 to 19% without a co-pack approach. The governments in the African markets chose to pursue a “product switching” strategy in which single units of ORS would be phased out and replaced with co-packs. Through this approach, governments aimed to rapidly increase combined coverage to match the portion of patients that were already receiving ORS.

When applying a co-pack strategy in the public sector, it is important to only introduce the co-pack when its price is equal to or below the combined price of single units. As an example, Uganda National Medical Stores issued a tender before there was strong local competition for quality co-packs; as a result, a bid was awarded in which the price of co-packs was higher than the full cost of treatment with individual units. The government renegotiated after competitive pricing was available. In countries like Nigeria and Kenya, the switch was made after there were affordable, quality co-packs on the market, leading to co-pack pricing that was even more favorable than the single units. In fact, in Nigeria, Uganda, and Kenya, the co-pack costs 36% less than single units in private outlets at the end of the programs (Table S2 in **Online Supplementary Document**).

### Provider demand

While the program targeted both public and private sector providers to improve the percent of pediatric diarrhea cases receiving the correct treatment, ORS and zinc coverage rates were higher among cases seeking care from public sector providers (average of 76% for ORS and 50% for zinc) compared to private sector providers (average of 55% for ORS and 33% for zinc). This finding was expected as there are often well-established platforms to reach public sector clinicians as well as more opportunity for management accountability. Other studies have also observed higher ORS coverage among cases seeking care in the public sector compared to private sector [40]. However, the overall patient impact of increased dispensing rates in public facilities is dependent on the relative portion of patients who are seeking care in public facilities.

### Consumer demand

To generate demand among caregivers, the program relied on quantitative and qualitative research to not only understand current practices but also what messages and strategies would be most effective in changing behavior. The demand efforts focused on measurable changes in *use* – not just awareness or knowledge. In India, program data revealed that mass media did in fact have penetration with rural households and also influenced coverage increases [32]. In India, the program conducted robust market research and tested messages and television spots with the target audience, rural mothers with children under five. The results of this research helped to inform the final messages, creative content of television and radio spots, and the selection of channels to maximize reach and exposure rates.

Not unexpectedly, the markets with the highest rates of care-seeking for diarrhea – 89% in India and 76% in Nigeria–also had the greatest coverage increases. India and Nigeria also had the greatest rates of private sector care-seeking (79% of those seeking care in India and 57% in Nigeria). More proximate access to care through an expansive network of private outlets may have contributed to the higher care-seeking rates, and thus higher coverage, in India and Nigeria. This potential advantage is particularly relevant for products like ORS and zinc that can be legally dispensed as over-the-counter products.

### Enabling environment

In all focal geographies, strong government leadership influenced the speed of implementation and the sustainability of results. For example, in Nigeria, the primary program interventions were anchored within Nigeria’s high-profile National Essential Medicines Strategy, and implementation was led by the government and managed through existing technical working groups at national and state levels. These coordinating mechanisms provided ongoing accountability for progress and also facilitated rapid problem solving across government, donors, and nongovernmental organization stakeholders. In Kenya, the central procurement authority issued a strong directive to all 47 county governments to recommend switching to a co-pack, and the Kenyan government also bundled single-units in advance of the co-pack introduction to prepare the system for the switch. These efforts likely helped to accelerate the switch to co-packs and the uptake of the combined treatment.

In some markets, broader trends with procurement of essential medicines may have influenced the results of the program as well. For example, in Kenya, procurement authority for the country was devolved from a centralized system to 47 individual counties during the course of the program. Through this period of transition, availability of essential medicines in public sector facilities, including ORS and zinc, was also strained. This broader change in Kenya’s national procurement approach likely contributed to lower gains than expected in the Kenya context [33].

In Uganda, government procurement of ORS had declined in the early part of the program which may have limited improvement in ORS coverage. Between 2011 and 2014, government procurement of ORS had declined by 64%. The decline coincided with the decentralization in the quantification of the Essential Medicine kit from the Central Medical Store to each district and the switch from single ORS sachets to a more expensive co-pack. A household survey conducted by the program at the end of 2014 found ORS coverage had declined from 44% in 2011 to 35% by 2014. By 2016, ORS coverage had increased to 47%.

### Study limitations

Our evaluation relies on triangulation of multiple data sources, including data collected primarily by the program and secondary data sources such as the DHS. A limitation of this methodology is that the use of multiple sources may affect comparability between data sources. While several efforts were made to adopt the sampling, survey, and implementation methods of the DHS, we had to adjust aspects of the methodology to fit into the available resources. Thus, we cannot rule out that the program household surveys may not be fully comparable to the DHS results. However, we have compared our survey results with other available data sources to validate the estimates obtained in our surveys. In India, we compared our estimates with those found in the National Family Health Survey (NFHS-4), which was conducted approximately a year prior to our surveys, and found similar results. In the NFHS-4, the pooled, weighted ORS coverage for Gujarat, Madhya Pradesh, and Uttar Pradesh was 42% and the combined ORS and zinc coverage was 16%. In CHAI’s endline household survey, we found pooled, weighted ORS coverage of 48% and combined ORS and zinc coverage of 19%. In Nigeria, we compared our estimates with those found in the MICS 2016-17 survey, which was conducted within nine months of our surveys. The pooled, weighted combined ORS and zinc coverage estimate for the eight program states was 25% in the MICS 2016-17 results while we found combined ORS and zinc coverage to be 30%. We did find higher coverage of ORS (55%) than the MICS 2016-17 (40%), which may be due to survey timing, sampling, or how the questions were asked. Our survey was focused exclusively on diarrhea and interviewers carried pictures of local ORS and zinc brands to improve recall, while the MICS survey covers an extensive range of child health and education questions. Going forward however, the DHS and MICS can be utilized to measure whether ORS and zinc coverage has sustained, and ideally increased.

Another limitation is that the pre-post evaluation methodology prevents us from estimating the attribution of the coverage changes to the program. Due to the program design which worked with and through the government for sustainability reasons, we do not think it is feasible to fully separate the program efforts directly funded by the program from government activities as these were often done jointly. The changes in coverage are likely a result of the combined efforts of the program, the government, and other partners.

We also acknowledge that the authors of this paper were part of the program design and implementation which may introduce inherent bias. Even though several authors were part of the implementing team, we are confident that our study design was rigorous and that an external evaluator would find the same results. We attempted to mitigate the bias by hiring an external research agency to oversee the data collection. As mentioned above, we have also compared our survey results with independent external data sources to validate the survey findings. Furthermore, in Uganda, the program relied entirely on the DHS 2011 and 2016 to evaluate the change in coverage. Fortunately, the program period (2012-2016) neatly aligned with the DHS surveys, thus providing a fully independent source for ORS and zinc coverage changes. The DHS surveys found significant change in combined ORS and zinc coverage from 1% to 30%.

Lastly, the lack of routine coverage surveys conducted in other geographic areas limited our ability to draw country comparisons, particularly during the specific program period. In the Asia/Middle East/Eastern Europe region, we were only able to find seven countries with two household surveys conducted between 2008 and 2016. The region may not be fully comparable to the conditions in the Indian program states. The comparison between our African program areas to the rest of Sub-Saharan Africa is also not ideal as Sub-Saharan Africa encompass a very diverse set of socioeconomic and political environments. However, the rates of ORS and zinc increase during the program period are much higher than that prior to the program period. Furthermore, the rate at which combined ORS and zinc coverage increased in Nigeria and Uganda are higher than even that of Bangladesh.

## CONCLUSION

Increasing ORS and zinc coverage at scale in high-burden countries and states is possible through a comprehensive approach that targets both demand and supply barriers, including pricing, optimal product qualities, provider dispensing practices, stocking rates and consumer demand.

Box 2 **Program summary and lessons learned in Kenya**

***Approach***: In Kenya, ~ 50% of children with diarrhea seek care outside the home, ~ 40% treat in the home, and ~ 10% take no action. Of those seeking care, nearly 2/3 do so in the public sector. Given this, the program approach aimed to change clinical management and dispensing practices in the public sector from ORS alone to both ORS and zinc through a co-pack strategy. In partnership with the MOH, the program also focused on educating mothers on diarrhea prevention and treatment though mass media, ORT corners, and health talks since over 90% of Kenyan mothers attend immunization visits for their child. In addition, the program engaged new, quality suppliers to enter the market by sharing credible demand forecasts, including the MOH’s co-pack approach. CHAI also provided suppliers with in-kind promotional materials for use in detailing visits. Five new co-pack suppliers entered the market, and retail prices declined 55%. In 20 of Kenya’s 47 counties, the program also supported county-level management teams to rollout IMCI in public facilities, including an offsite 5-day training followed by routine CMEs and supportive supervision.

***Mistakes/Challenges:*** The greatest challenge in Kenya was the devolution of the health system from one central procurement authority (KEMSA) to 47 different counties in 2014. Forecasting, quantification and ordering became the responsibility of county governments for the first time, causing challenges for consistent stocking of all essential medicines, including ORS and zinc.

***Critical Success Factors:***

- Active, decisive government leadership at national /county level – high priority & willingness to commit domestic finances
- Strong private sector suppliers with existing sales teams that responded to market opportunity to meet growing demand
- Caregivers generally more educated, trust public sector

Box 3 **Program summary and lessons learned in program states in Nigeria**

***Approach***: In the 8 program states (Lagos, Kano, Rivers, Bauchi, Cross River, Kaduna, Katsina, and Niger), ~ 75% of pediatric diarrhea cases seek care, with 25% going to the public sector and 50% to the private sector (DHS 2013). At the start of the program, zinc/ORS prices were high ($1.55 per course), and there was only one quality supplier of low osmolarity ORS and none for pediatric zinc dispersible tablets. The program provided TA to local manufacturers on market intelligence, formulation development, local registration, and cost reduction strategies. From these efforts, 10+ quality products, including six co-packs, were introduced in the market. To target private providers, the program worked with the Pharmacy Council of Nigeria (PCN) and the National Association of Proprietary Patent Medicine Vendors (NAPPMED) to reach >90% of registered PPMVs with peer detailing. To increase rural stocking, zinc/ORS promotional kiosks were placed at wholesale distribution points, and the program partnered with local sub-distributors with significant rural footfall to expand the program’s reach. In the public sector, over 85% of providers were reached through routine training and mentoring. The program also supported the state and national government on forecasting and quantification and on designing and implementing Nigeria’s 2012 National Essential Medicines Scale up Strategy. Consumer demand was driven through facility health talks, female vanguard groups, religious schools and a radio campaign.

***Mistakes/Challenges:*** Efforts to incentivize national suppliers to meet rural availability and price targets failed; despite strong buy-in, suppliers lacked expertise in rural markets and the drive to make meaningful investments in novel business models to reach these segments as their existing urban market share bred risk aversion.

***Critical Success Factors:***

- Strong government leadership at national/state level – high priority and willingness to commit domestic finances
- Strong PPMV association provided platform for timely and cost-effective message dissemination

## Additional material

Online Supplementary Document
