# Supplementary material for: Increasing coverage of pediatric diarrhea treatment in high-burden countries
Source: J Glob Health. 2019 Apr 27;9(1):0010503. doi: 10.7189/jogh.09.010503 (PMC6513503; doi:10.7189/jogh.09.010503)
Supplement: Online Supplementary Document [file jogh-09-010503-s001.pdf]

J Glob Health 2019; 9: 010503

Online Supplementary Document

Schroder et al. Evaluation of a comprehensive program model to increasing coverage of pediatric diarrhea treatment in high-burden countries

Contents

Figure S1. Program theory of change 2

Appendix S1. Public facility audit and private outlet survey methodology 3

Appendix S2. Diarrhea deaths averted 4

Table S1. Lives saved estimates 2012-2016 5

Figure S2. Availability of ORS from public sources 6

Figure S3. Availability of zinc from public sources 6

Figure S4. Availability of ORS in private outlets 7

Figure S5. Availability of zinc in private outlets 7

Figure S6. Cost of full treatment with ORS and zinc (in USD) 8

Table S2. Cost of full treatment with ORS and zinc by end of program (in USD) 8

Figure S1. Program theory of change

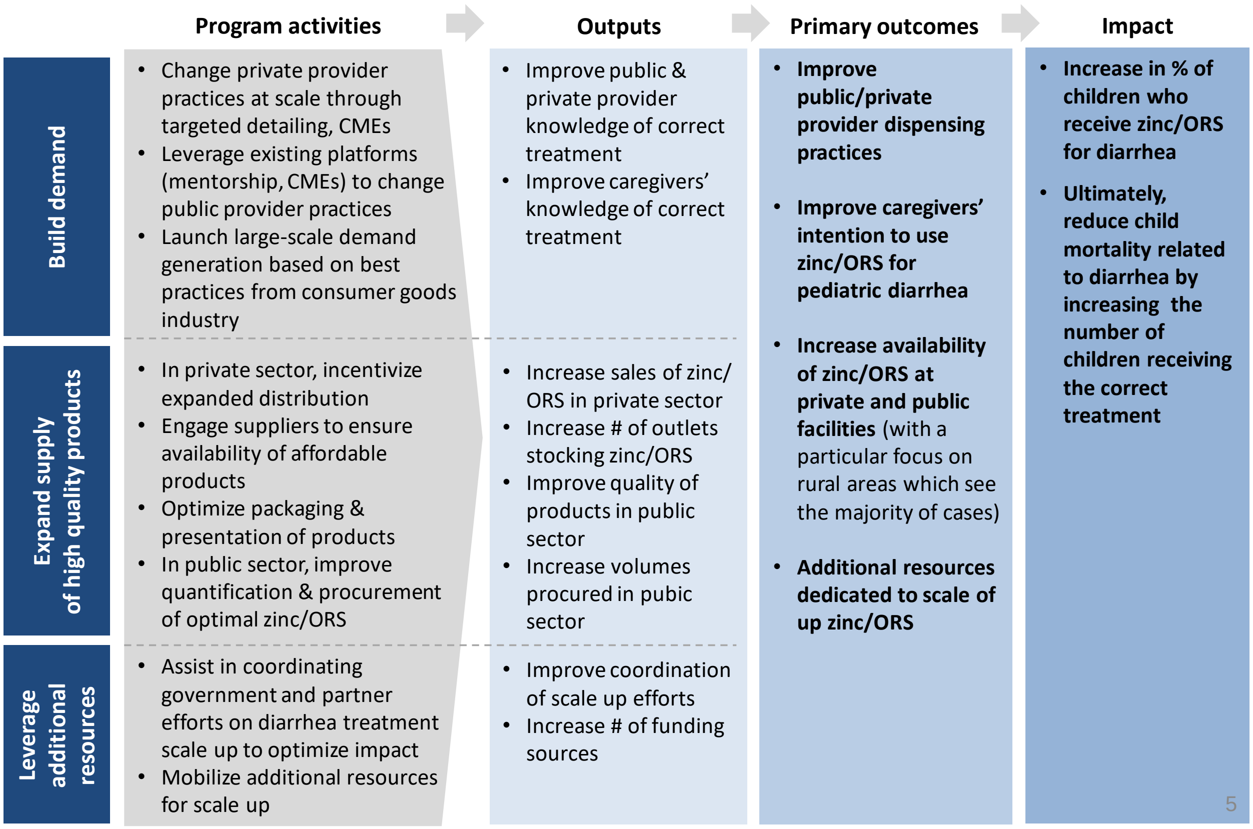

Appendix S1. Public facility audit and private outlet survey methodology

In India, Nigeria, and Uganda, public facility audit surveys and private outlet surveys were conducted at baseline, midline, and endline of the program. In Kenya, only private outlet surveys were conducted at two periods: baseline and midline.

Public facilities and private outlets were sampled using two-stage cluster randomized selection. The clusters were census enumeration areas in India, Nigeria, and Uganda, while in Kenya, the clusters were ward administrative areas. In the first stage, the study used the most recent census as the sampling frame (i.e. the 2011 Indian national census, 2002 Ugandan national census, and 2006 Nigerian national census). In the second stage, trained enumerators visited each cluster and interviewed community members to identify all public and private sources of care. Public sources of care included community health workers, dispensaries, health centers, and hospitals. Private outlets included any private source where community members living in the cluster could obtain medicines and most often included pharmacies, registered and unregistered drug shops, and formal and informal private healthcare practitioners. Once an exhaustive list of public facilities and private outlets which serve the community living in the cluster was developed, the trained enumerators visited each facility and outlet to conduct an audit to determine whether ORS and zinc were stocked at the outlet on the day of the survey. In private outlets, enumerators also asked about the prices of ORS and zinc.

Appendix S2. Diarrhea deaths averted

We use the Lives Saved Tool in Spectrum version 5.51 to estimate the number of diarrhea deaths averted due to ORS and zinc scale-up between 2012 and 2016 in each of the program geographies. We first generate population projections for the program years. For Kenya and Uganda, we use the default national population projections. For Nigeria, we downloaded the state population projections for the 8 program states from the Lives Saved website[1]. For India, we generated population projections for Gujarat, Madhya Pradesh, and Uttar Pradesh using the 2011 Indian national census and the 2015-16 NFHS state results.

We enter ORS and zinc coverage estimates obtained from the household surveys in the year the survey was conducted and linearly interpolate coverage estimates between the baseline and endline survey years. Where possible, we also updated all other intervention coverage estimates (e.g. vaccinations, bed net coverage, etc.) though this was only possible in India and Uganda where the NFHS and DHS results were available at the end of the program and contained coverage estimates for other interventions. In these instances, we update those coverage estimates for the year the survey was conducted and interpolate coverage figures between the previous survey and the most recent NFHS and DHS. Otherwise, we kept other intervention coverage estimates at default values.

Finally, we generate additional lives saved using 2012 as the reference period and sum all diarrheal deaths averted between 2012 and 2016.

[1] Nigeria state population projections downloaded from <http://livessavedtool.org/index.php/all-downloads-tools#Subnational> on June 23, 2017.

Table S1. Lives saved estimates 2012-2016

Program area	Estimated lives saved (sensitivity bounds*)
India (3 states[1])	48,030 (38,590 – 56,090)
Kenya	3,340 (2,670 – 3,920)
Nigeria (8 states[2])	18,160 (14,810 – 20,920)
Uganda	6,560 (4,620 – 8,210)
Total	76,090 (60,690 – 89,140)

*Sensitivity bounds are calculated using the Lives Saved Tool and based upon the highest level of effectiveness reported (upper bound) and the lowest levels of effectiveness reported (lower bound)

Figure S2. Availability of ORS from public sources

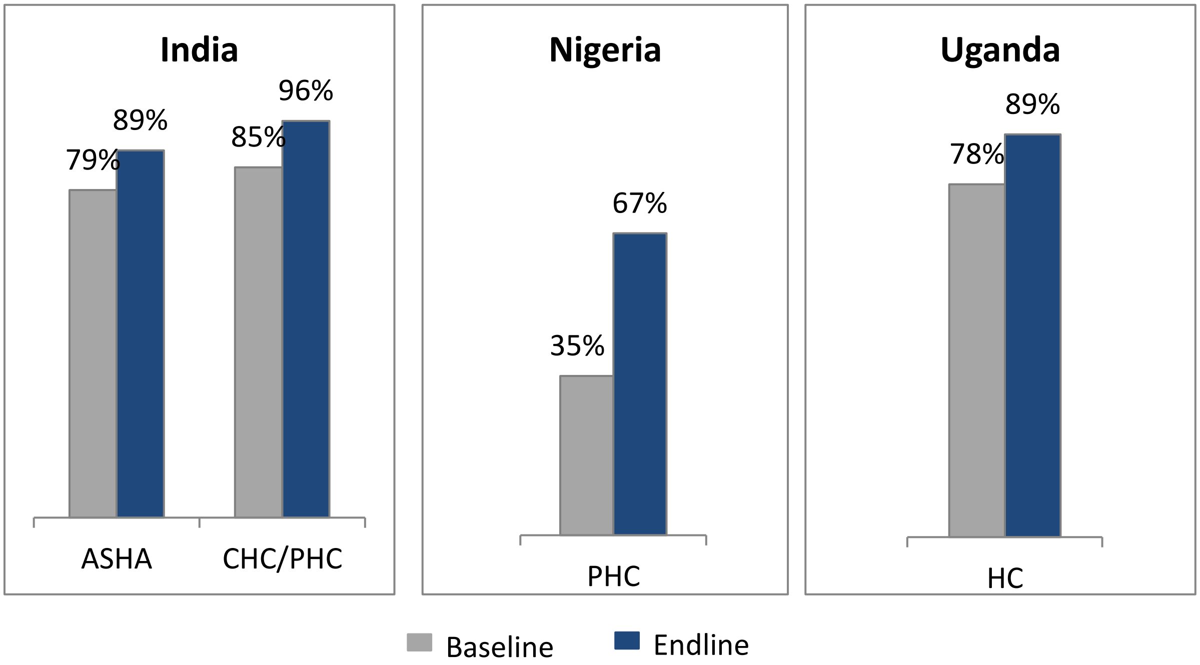

Figure S3. Availability of zinc from public sources

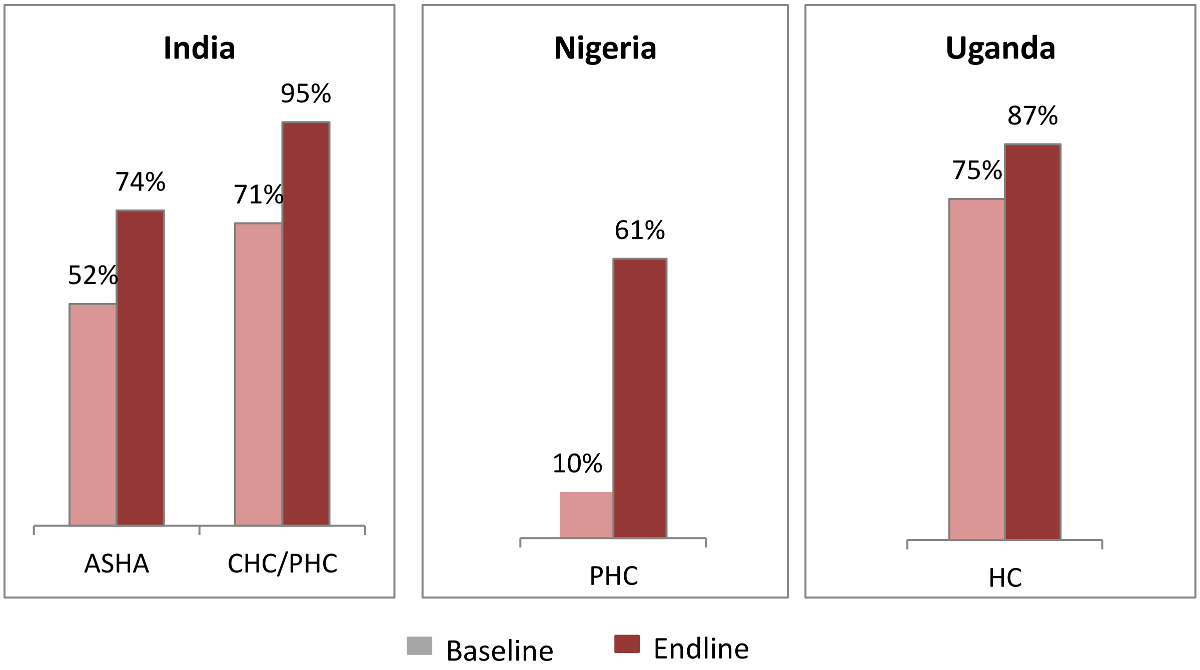

Figure S4. Availability of ORS in private outlets

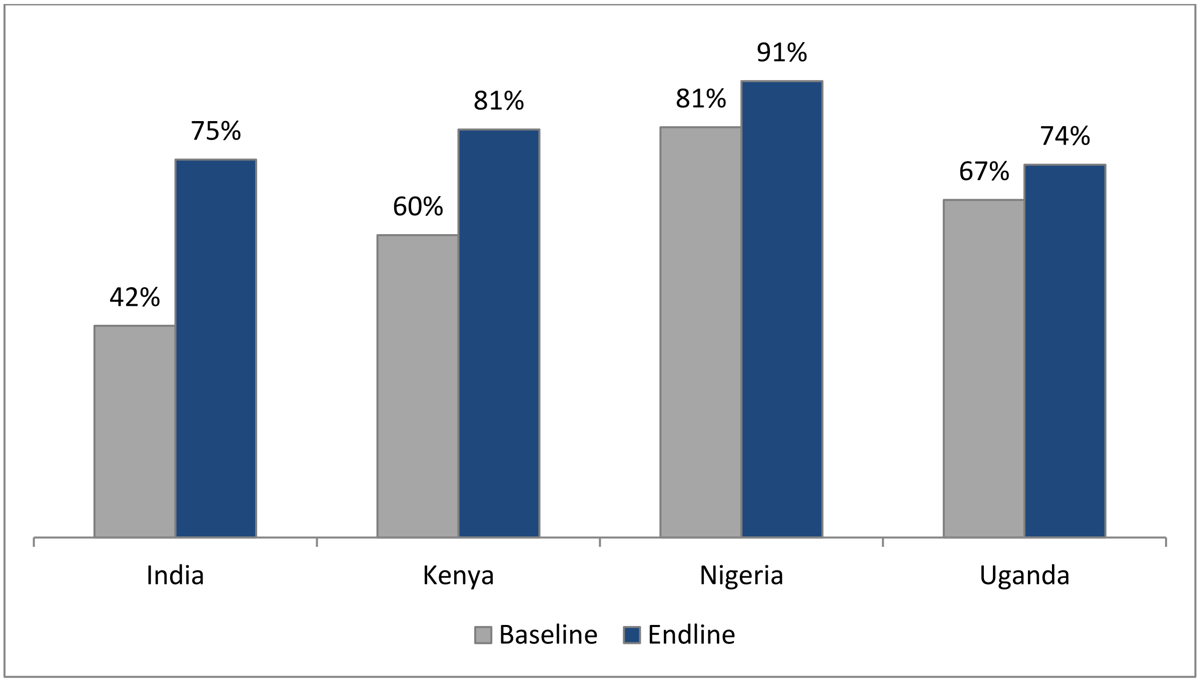

Figure S5. Availability of zinc in private outlets

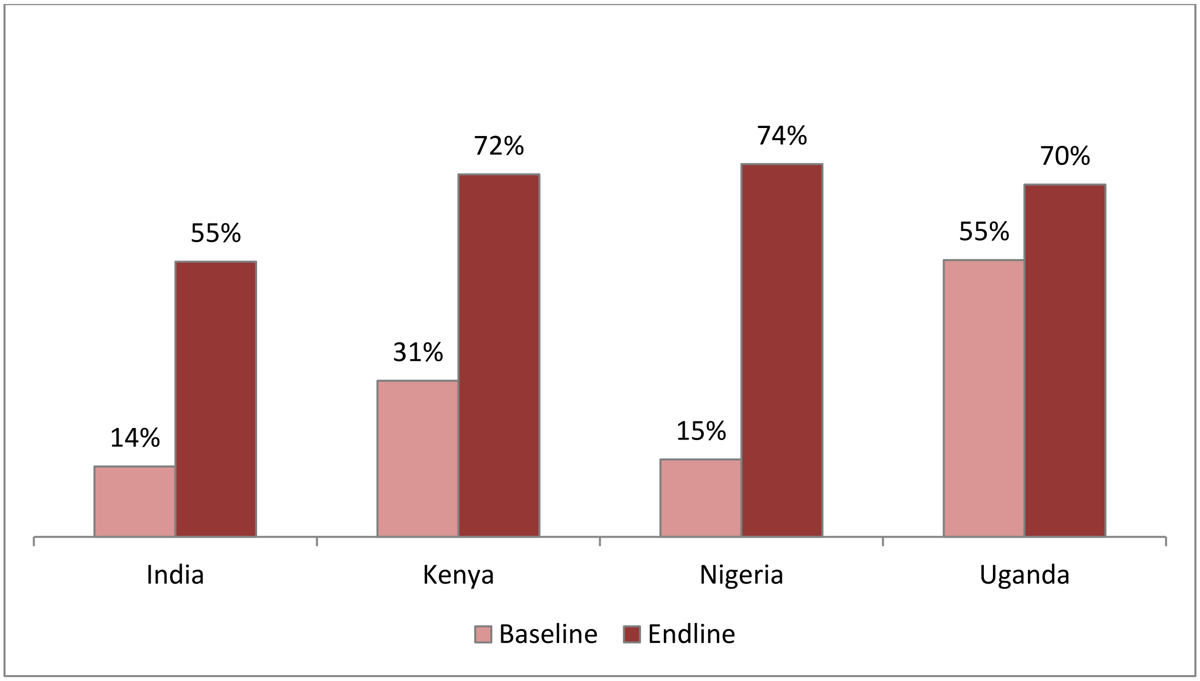

Figure S6. Cost of full treatment with ORS and zinc (in USD)

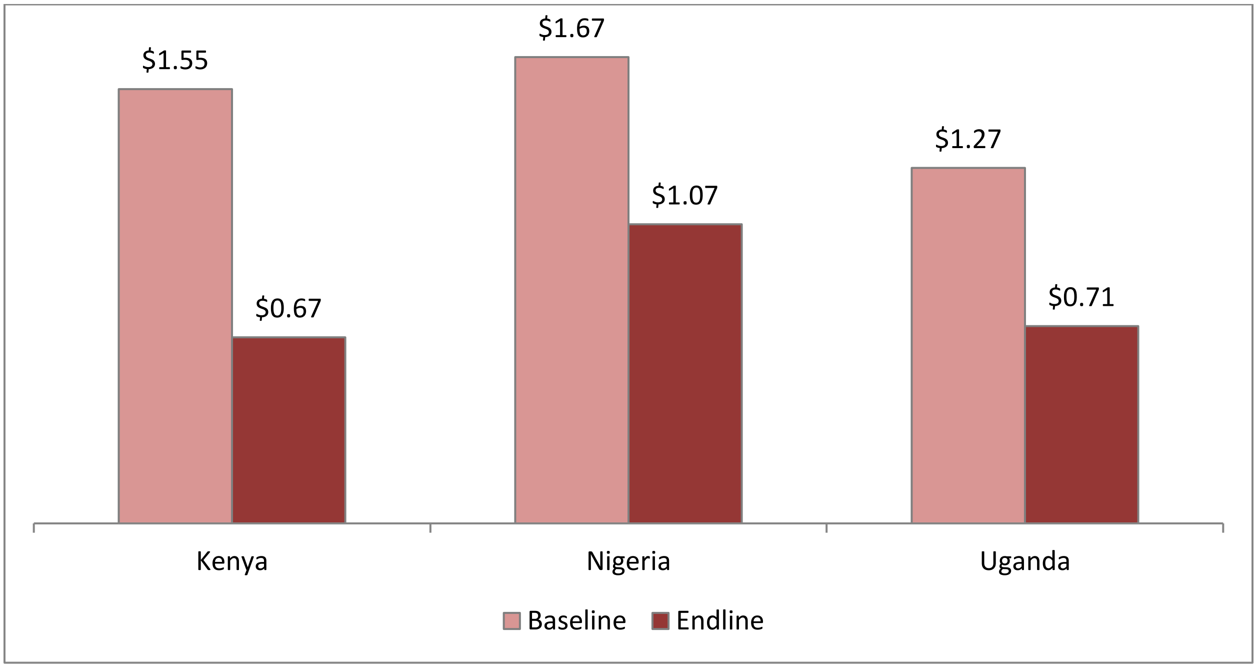

Table S2. Cost of full treatment with ORS and zinc by end of program (in USD)

Country	Single treatments (2L equivalent of ORS and 10 zinc tablets)	Co-pack (2L equivalent of ORS and 10 zinc tablets)
Kenya	$1.55	$0.67
Nigeria	$1.23	$1.07
Uganda	$1.06	$0.71
